# Intellectual Disability Profiles, Quality of Life and Maladaptive Behavior in Deaf Adults: An Exploratory Study

**DOI:** 10.3390/ijerph19169919

**Published:** 2022-08-11

**Authors:** Johanna Eisinger, Magdalena Dall, Jason Fogler, Daniel Holzinger, Johannes Fellinger

**Affiliations:** 1 Research Institute for Developmental Medicine, Johannes Kepler University, 4020 Linz, Austria; 2 Division of Developmental Medicine, Boston Children’s Hospital, Boston, MA 02115, USA; 3 Leadership Education in Neurodevelopmental and Related Disabilities/Institute for Community Inclusion (LEND/ICI), Boston Children’s Hospital, Boston, MA 02115, USA; 4 Institute of Neurology of Senses and Language, Hospital of St. John of God, 4020 Linz, Austria; 5 Institute of Linguistics, University of Graz, 8010 Graz, Austria; 6 Division of Social Psychiatry, University Clinic for Psychiatry and Psychotherapy, Medical University of Vienna, 1090 Vienna, Austria

**Keywords:** intellectual disability, deaf, adaptive behavior, intellectual functioning, domain discrepancy, maladaptive behavior, quality of life

## Abstract

Individuals who are prelingually deaf and have intellectual disabilities experience great challenges in their language, cognitive and social development, leading to heterogeneous profiles of intellectual and adaptive functioning. The present study describes these profiles, paying particular attention to domain discrepancies, and explores their associations with quality of life and maladaptive behavior. Twenty-nine adults with prelingual deafness (31% female) and mild intellectual functioning deficits (mean IQ = 67.3, SD = 6.5) were administered the Vineland Adaptive Behavior Scales-II (VABS-II) and an adapted sign language version of a quality of life scale (EUROHIS-QOL 8). Intellectual disability domain discrepancies were characterized as at least one standard deviation difference between the social domain and IQ and the practical domain and IQ, and a significant difference, according to the VABS-II manual, between the social and practical domains. Domain discrepancies were found between intellectual functioning and both the practical (58.6%) and social domain (65.5%). A discrepancy between intellectual and social functioning was significantly associated with a higher level of internalizing maladaptive behavior (T = 1.89, *p* < 0.05). The heterogeneous profiles highlight the importance of comprehensive assessments for adequate service provision.

## 1. Introduction

Deafness is a heterogeneous condition that can impact communication, social–emotional development and cognitive development [1]. Around 7 per 10,000 people have severe to profound hearing loss, with onset before language acquisition [2,3]. 

Approximately one-third to one-half of individuals who are prelingually deaf or hard of hearing have additional disabilities [4,5], most commonly intellectual disability [6]. Additive deprivation of language and communication, stemming from delayed identification, insufficient or late provision of hearing technology and little or no access to sign language, further impedes these individuals’ community participation [7,8,9,10]. 

The diagnostic criteria for intellectual disability have been revised in the Diagnostic and Statistical Manual of Mental Disorder-Fifth edition (DSM-5; [11]) to encourage a more comprehensive patient assessment, with greater weight given to adaptive functioning than intellectual functioning for the purpose of ascribing intellectual disability severity [11]. Whereas intellectual functioning generally involves abilities such as reasoning, problem solving, knowledge and experience [12], adaptive functioning refers to the skills that are learned and performed to meet the everyday demands of one’s community or society [13], suggesting that adaptive behavior may be the more malleable (and hence important) intervention target to unlock an individual’s full potential. Adaptive functioning includes three domains: the conceptual domain, including applied skills in language, reading, writing, math, reasoning, knowledge and memory; the social domain, referring to empathy, social judgment, interpersonal communication skills and the ability to make and retain friendships; and the practical domain, including self-management in areas such as personal care, job responsibilities, money management, recreation and organizing school and work tasks [11].

Intellectual and adaptive functioning, the two aspects of intellectual disability, are related but separate constructs [13]: a large meta-analysis of 148 samples containing a total of 16,468 participants showed a moderate relationship (r = 0.51) between intelligence and adaptive behavior, which is stronger in lower IQ groups [14].

With this more nuanced definition of adaptive functioning has come greater interest in intellectual disability domain discrepancy, in which one domain is markedly more deficient than another, as well as inquiry into whether different populations have unique, or at least specific, intellectual disability profiles. Sparrow, Cicchetti and Balla [15], authors of the Vineland Adaptive Behavior Scales-II (VABS-II), provide various adaptive functioning profiles based on pairwise comparison of the four adaptive behavior domains (communication, socialization, daily living skills and motor skills) outlined in the Vineland-II manual. When comparing the specific profile of individuals with hearing impairment with samples matched by age range and controlled for sex, ethnicity and education level, the researchers found that individuals with hearing impairment had lower levels of communication and daily living skills than the IQ-matched sample with typical hearing. The socialization scale appeared as a relative strength, though still lower than the non-clinical group [15]. 

There is a growing body of research on adaptive profiles in individuals with different neurodevelopmental disorders [16,17,18,19,20,21,22]. Tillmann et al. [23] examined how IQ and levels of ASD symptom and autistic trait severity are associated with adaptive functioning and suggested that core ASD-related social communication problems contribute both to adaptive functioning impairments and to the discrepancy between IQ and adaptive functioning. Further supporting this point, a discrepancy between intellectual functioning and adaptive skills was found to be significantly correlated with depression and anxiety in a sample of adults with ASD without intellectual disability, in which socialization was by far the largest weakness [24].

Studies correlating adaptive profiles with such clinically relevant variables as quality of life (QOL) and problem behavior (e.g., [13,25]) show divergent results. Tassé [13] and Simoes et al. [26] found a positive correlation between adaptive behavior and QOL in samples of individuals with mild-to-moderate intellectual disability, whereas Graves et al. [18] did not find significant associations between adaptive functioning and self-reported QOL in a sample of adults with Down syndrome. Jones et al. [27] found higher levels of problem behavior to be associated with more severe degrees of intellectual disability. Curiously, Balboni et al. [25] found that a subgroup of individuals with intellectual disability with the highest levels of problem behavior also had higher levels of adaptive behavior, explaining that a basal level of adaptive skills appears to be necessary for the person to be able to engage in their environment, positively or negatively. 

No research to date has investigated the intellectual disability profiles and the relationships between intellectual disability domain discrepancies, QOL and maladaptive behavior in a population with prelingual deafness and intellectual functioning deficits. Hence, the main aim of this study is twofold: (a) to describe the intellectual disability profiles and potential intellectual disability domain discrepancies in a sample of adults who are deaf with borderline and mild cognitive functioning impairment and (b) to explore how these intellectual disability profiles and domain discrepancies are related to maladaptive behavior and self-reported QOL in this population. We explored whether expressed intellectual disability domain discrepancies between cognitive potential and lower social and practical abilities are experienced as stressful barriers to unlock one’s potential and therefore may be linked with lower quality of life and increased rates of maladaptive behavior. 

## 2. Materials and Methods

### 2.1. Participants

This cross-sectional exploratory study was conducted within three therapeutic living communities (Lebenswelt) specifically developed to accommodate the needs of individuals with deafness and additional disabilities, focusing on supporting communication, social relationships, conflict resolution and work satisfaction. They are characterized by the constant use of sign language; one-quarter of the staff members are deaf themselves [7].

We recruited 29 individuals (9 women and 20 men) who met the inclusion criteria of having at least moderate hearing impairment and an IQ score between 50 and 85 (see Table 1). Of these participants, 93% were profoundly deaf and 7% had moderate hearing loss. Nearly all the participants joined their therapeutic communities with lifetime histories of potentially traumatic events—a sadly common finding among members of the deaf community [28,29]—and about 38% had experienced at least one depressive episode [30]; however, no participant was experiencing an active depressive episode during the time of data collection. Their length of enrollment in the therapeutic living communities ranged from 6 months to 20 years. Most of the participants (n = 23; 79.3%) lived and worked in these communities, and the remaining participants (n = 6; 20.7%) only took part in the workshop facilities. Their mean age was 46.89 years (SD = 16.42, range 20–73 years), and their mean IQ score was 67.31 (SD = 6.49, range 57–82). Based on the ICD-10/WHO criteria, the majority (72.4%) were classified as having mild deficits indicated by an IQ score between 50 and 69 [31]. In addition, half (51.7%) of the participants were currently diagnosed with intellectual disability with challenging behavior (F70.1 (ICD-10)), 20.7% with cerebral palsy, 13.8% with epilepsy and 13.8% with autism. Table 1 displays the sample characteristics in detail.

This study was approved by the ethical committee of the hospital St. John of God in Linz, Austria. Consent was given by the participants themselves and/or by their legal guardians (if applicable).

### 2.2. Instruments

#### 2.2.1. Intellectual and Adaptive Functioning

**Figure 1:**
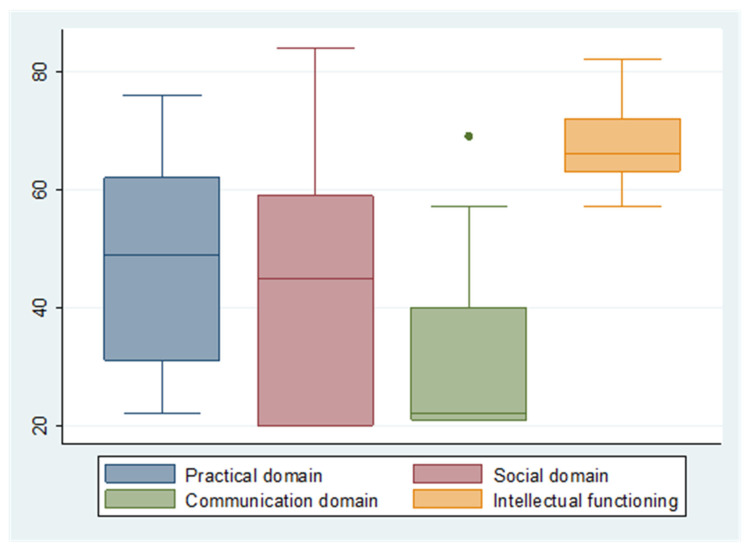
Boxplots of the domains of intellectual disability (standard scores of Vineland-II domains and SON-R 6-40).

The Vineland Adaptive Behavior Scales-II [15] is a comprehensive measure of adaptive behavior. This standardized norm-referenced assessment instrument provides information on an individual’s adaptive behavior from birth to 90 years of age across motor, communication, daily living and socialization skills. A standard score (M = 100, SD = 15) for each domain is calculated as well as a summary adaptive behavior composite score. The subscales of the Vineland-II do not perfectly align with the current tripartite model of adaptive behavior described in the DSM-5′s definition of intellectual disability. Tassé and Mehling [12] proposed the following alignment or “cross-walking” of the VABS-II domains with the three domains of adaptive functioning identified in DSM-5: communication = conceptual skills; socialization = social skills; and daily living skills = practical skills. Accordingly, the socialization and daily living skills subscales of the Vineland-II were used to address the social and practical domains. However, two of the three sections of the communication subscale relate to the comprehension and production of spoken language. After adapting comprehension items to a visual modality, many items specifically referring to the structure of spoken language (e.g., intonation, verb inflection, prepositions, pronunciation, pronouns) had to be replaced by Austrian sign language items estimated to be functionally equivalent and of a comparable level of complexity. This non-validated adaptation resulted in significant floor effects that strongly weighed against its being considered for measuring the conceptual domain in this group of adults who are deaf with intellectual disability (see Figure 1).

To assess participants’ intellectual functioning, we administered the Snijders-Oomen Non-verbal Intelligence Scale for individuals (SON-R 6-40; [32]). The SON-R 6-40 assesses the participant’s non-verbal cognitive developmental level and provides a standard IQ score (M = 100, SD = 15), making it relatively easy to compare with the standard scores derived from the two VABS-II subscales.

#### 2.2.2. QOL

The EUROHIS-QOL 8-item index (European Health Interview Surveys) is a short version of the WHOQOL-BREF. It consists of 8 questions that are also included in the 26 questions of the WHOQOL-BREF [33]. All four domains (physical, psychological, social and environmental) are represented, each with two questions. A normative study in Germany showed good construct validity and reliability [34]. An adapted, easy-to-understand sign language version of the EUROHIS-QOL 8-item index was administered to assess participants’ self-rated QOL [35]. Fellinger et al. (2021) [35] demonstrated that reliable and valid self-reports of QOL can be obtained from adults who are deaf with mild-to-moderate intellectual disability using standard inventories such as the EUROHIS-QOL adapted to the linguistic and cognitive levels of these individuals. The EUROHIS-QOL 8-item index score was computed as the mean score across the eight items, ranging from 1 (worst QOL) to 5 (best QOL). The test–retest reliability was good (0.75), and internal consistency showed a Cronbach’s Alpha of 0.78.

#### 2.2.3. Maladaptive Behavior

The VABS-II includes two subscales for internalizing and externalizing maladaptive behavior. The two subscales are reported as v-scale scores. A v-scale score below 18 indicates a non-clinical level of maladaptive behavior; a score between 18 and 20 indicates an elevated level; and a score between 21 and 24 indicates a clinically significant level of maladaptive behavior [15].

### 2.3. Data Collection

Data collection took place between September 2017 and March 2018. Participants’ clinical characteristics (i.e., type and degree of hearing loss; psychiatric, behavioral and neurological diagnoses) were extracted from the medical records of the individuals at the hospital St. John of God. The QOL self-reports were gathered through a structured interview between the resident and a sign language-competent staff member who was not directly involved in the care of the residents (non-involvement in direct care was considered important in order for the participants not to feel pressured to give answers that they thought would be preferred by the interviewer).

VABS-II data were collected for each participant by the staff psychologist in consultation with either a family member or the participant’s primary caregiver. Primary caregivers serve the roles of coach, case manager, advocate and personal assistant for residents and were therefore thought to be particularly well-qualified to serve as informants for this study.

### 2.4. Computing Intellectual Disability Domain Discrepancies

Within the context of this paper, we use the term *intellectual disability domain discrepancy* to indicate that there is a substantial difference between the level of intellectual, social and/or practical adaptive functioning. For the purpose of this study, when comparing intellectual functioning with the social or practical domain, a difference of at least 15 points (=1 SD) between the IQ score (SON-R 6-40) and the socialization and daily living skills (DLS) standard scores indicates an intellectual disability domain discrepancy. When comparing the social and practical domains, a difference between the socialization and the DLS standard scores with a significance level of 0.05 according to the VABS-II manual indicates a domain discrepancy between these two domains.

### 2.5. Statistical Analysis

First, univariate analysis of the key variables was applied to describe intellectual disability domains, QOL and maladaptive behavior, as well as domain discrepancies. Next, Spearman’s correlation was performed to test for a correlation between intellectual disability domains (social, practical and intellectual functioning), QOL and maladaptive behavior. To investigate whether there were significant differences in the means of self-reported QOL and internal and external maladaptive behavior with and without intellectual disability domain discrepancies, an independent samples *t*-test was performed.

## 3. Results

### 3.1. Intellectual Disability Domains, QOL and Maladaptive Behavior

Figure 1 shows the distribution of the median standard scores of (a) SON-R 6-40 intellectual functioning and (b) the three domains of Vineland-II adaptive functioning (practical, social and communication). Intellectual functioning emerged as the strongest domain, with a mean standard score of 67.31 (SD = 6.49) and no standard scores lower than 57 (see Table 2). The individuals also demonstrated a low level of practical functioning with a mean score of 48.97 (SD = 17.41), with more than half of the sample classified as having moderate or severe deficits. The social domain was the weakest domain with a mean score of 41.45 (SD = 19.66), indicating a moderate level of impairment according to the classification of the ICD-10 [31], and 12 (41.4%) individuals can be classified as having severe deficits in this domain. The communication domain of the Vineland-II, with a median standard score of 22, emphasizing the floor effect described earlier, was impossible to use as a proxy for the conceptual domain.

### 3.2. Correlations between the Intellectual Disability Domains and QOL and Maladaptive Behavior

Table 3 shows the Spearman’s correlation coefficients between the three domains. The social and practical domains are significantly positively correlated (r = 0.783, *p* = 0.000), as are the practical domain and intellectual functioning (r = 0.453, *p* < 0.05). The social domain and intellectual functioning are not significantly correlated (see Table 3). There are neither significant correlations between adaptive behavior and self-reported QOL nor significant relationships between adaptive behavior and maladaptive behavior, although correlations with social functioning approached the trend level of significance (*p* < 0.1).

### 3.3. Intellectual Disability Domain Discrepancies

Almost two-thirds of the individuals had an intellectual disability domain discrepancy between their intellectual functioning level and the social domain (n = 19, 65.5%), and more than half had an intellectual disability domain discrepancy between their intellectual functioning and the practical domain (n = 17, 58.6%). About one-quarter of the individuals had an intellectual disability domain discrepancy between the social and the practical domains (n = 7, 24.1%), where in all cases the social domain was the weaker domain. Thus, participants’ social adaptive skills were often poorer than their intellectual functioning and practical adaptive skills.

### 3.4. Associations between Intellectual Disability Domain Discrepancies and Self-Reported QOL as Well as Maladaptive Behavior (Independent Samples t-Tests)

When comparing participants with and without intellectual disability domain discrepancies, high mean QOL was endorsed across both groups, and no significant differences were found (see Table 4). Participants with a discrepancy between intellectual functioning and social domain had significantly higher levels of internalizing maladaptive behavior than the other groups (T = 1.889, *p* < 0.05).

## 4. Discussion

The aim of this study was to describe profiles of intellectual disability and domain discrepancies in a sample of adults who are prelingually deaf with mild and borderline cognitive impairment and to explore how these domains of intellectual disability are related with each other and associated with self-reported QOL and maladaptive behavior. Our findings provide a first indication of possible intellectual disability domain discrepancies among individuals with deafness and intellectual disability and highlight the value—as well as potential challenges or limitations—of DSM-5′s definition of intellectual disability for the deaf population. Furthermore, we investigated differences between those with and without domain discrepancies with respect to QOL and internalizing and externalizing maladaptive behavior.

Heterogeneous intellectual disability profiles were highly common among our participants, with only 24% showing no discrepancy between intellectual and adaptive functioning. In nearly two-thirds of the sample, intellectual disability domain discrepancies could be observed between intellectual functioning (65.5%) and both the practical (58.6%) and the social domain (65.5%). Domain discrepancy between the practical and social domain occurred in about one-fourth of cases (24.1%). Less than half of the sample had adaptive functioning levels in the practical and social domains that corresponded to their level of mild intellectual impairment, whereas severe levels of impairment were evident in the practical domain in 28% of the sample and in the social domain in 41%.

In our sample, the results of the communication subscales of the VABS-II, which were adapted but not originally designed for use in deaf populations, indicated severe deficits in 65.5% of the sample. Due to pronounced floor effects, the values of this domain were used neither as an equivalent for the conceptual domain nor for further calculations. Language is an important driver of acquiring social and practical skills above a rudimentary basal level [36], and the huge discrepancies seen in our sample with cognitive impairments may be due to the force multiplier effect of communication deprivation on the development of adaptive skills in this vulnerable population. These findings underscore how severe early childhood language deprivation impacts communication skills [10], even in our population where great effort has been taken to optimize access to communication through sign language in adult life. Other research in individuals with intellectual disabilities could show a strong association between communicative competences and QOL [37], which highlights the importance of access to language and communication. In contrast to the findings of Sparrow et al. 2005, in the present study with a sample who is prelingually deaf with mild cognitive deficits, the socialization domain appeared to be the weakest [15].

The relationship between intellectual functioning and the practical domain in our sample was moderately significant (r = 0.453, *p* < 0.05) and in line with the results of the meta-analysis of Alexander and Reynolds [14], whereas no significant correlations between intellectual functioning and the social functioning domain could be found in our sample.

Tassé [13] and Simoes et al. [26] both found a positive correlation between adaptive behavior and QOL in samples with mild-to-moderate intellectual disability. A similar effect is hinted at in our sample, with correlations trending toward statistical significance between social functioning and self-reported QOL (r = 0.354, *p* < 0.1), as well as a negative correlation between social functioning and internalizing maladaptive behavior (r = −0.351, *p* < 0.1). Conversely, neither intellectual functioning nor adaptive functioning in the practical domain were correlated with QOL or maladaptive behavior, a comparable finding to that observed by Graves et al. [18], who found no correlation between adaptive behavior and quality of life in adults with Down syndrome.

Having a statistically significant adaptive domain discrepancy between intellectual and social functioning was significantly correlated with higher levels of internalizing maladaptive behavior, a phenomenon that has also been observed in Autism Spectrum Disorder (e.g., [24,38]). Pending replication, one is tempted to query whether social connection is the critical ingredient in the positive adjustment and emotional well-being of individuals who are deaf (see, e.g., [39,40]), as well as individuals who are deaf and have intellectual disabilities, and we will pursue and welcome further inquiry in this area.

### Limitations

We must also note this study’s limitations. First and foremost, this is a small sample drawn from a highly enriched therapeutic residential care setting for adults who are deaf and have intellectual disabilities. Since this sample has only borderline-to-mild cognitive impairment, we make no claim to generalizability to the larger population of adults who are deaf with intellectual disability; much larger replication trials are needed.

Second, we must query whether the VABS-II, widely regarded as the “gold standard” adaptive measure in the majority of cases, is the most appropriate measure for this population. We therefore encourage further research in the service of formulating an optimal assessment battery both to gauge and, we hope, discover how to unlock these individuals‘ full potential. We would emphasize the importance of developing and validating a communication scale that is independent of (or less conflated with) solely spoken and auditory modalities.

## 5. Conclusions

Clearly, the assessment of intellectual disability transcends IQ, and we hope to inspire efforts toward an even higher level of measure refinement and collaborative research between investigators and participants. Underscoring the DSM-5′s incorporation of adaptive functioning into its definition of intellectual disability, our population of participants who were prelingually deaf with mild cognitive impairments had a broad array of strengths and challenges. Intellectual functioning emerged as a relative strength, whereas almost half our participants had severe deficits in the social domain. Critically, a higher level of internalizing maladaptive behavior was observed in those participants with a domain discrepancy between their intellectual and social functioning. We must acknowledge that, even with our best efforts in providing accessible therapeutic communities in adulthood, deficits in the social domain could not be fully compensated after histories marked by severe trauma and deprivation. This finding constitutes a strong case for the early prevention of communicative and social deprivation by providing full access to spoken and/or signed communication. Nevertheless, nuanced measurement of adaptive skills gives us a good opportunity to identify and target malleable factors to improve QOL in individuals who are deaf and have intellectual disability more broadly.

## Figures and Tables

**Table 1:** Sample characteristics.

Characteristics	N	%	Mean	SD	min	max
**Lebenswelt**						
Full program (residential and vocational)	23	79.31				
Day program/workshops only	6	20.69				
**Sex**						
Male/Female	20/9	68.97/31.03				
**Age**			46.90	16.421	20	73
**Hearing status**						
Moderate hearing loss (40–69 db)	2	6.90				
Profound hearing loss and deafness (>70 db)	27	93.10				
**Co-occurring disorders**						
Autism	4	13.79				
Epilepsy	4	13.79				
Cerebral palsy	6	20.69				
Intellectual disability with challenging behavior (F70.1 ICD-10)	15	51.72				
Lifetime depressive episodes	11	37.93				

**Table 2:** Descriptive results for intellectual disability domains, QOL and maladaptive behavior.

Intellectual Disability Domains, QOL and Maladaptive Behav	N	%	Mean	SD	min	max
**Levels of intellectual functioning impairments (based on SON-R 6-40)**	29		67.31	6.492	57	82
Borderline (standard score 70–84 according to ICD-10)	8	27.59				
Mild (50–69)	21	72.4				
**Levels of adaptive functioning impairments**						
Social domain (based on Vineland-II socialization)	29		41.45	19.66	20	84
Borderline (standard score 70–84 according to ICD-10)	1	3.45				
Mild (50–69)	12	41.38				
Moderate (35–49)	4	13.79				
Severe (20–34)	12	41.38				
Practical domain (based on Vineland-II DLS)	29		48.97	17.41	22	76
Borderline (standard score 70–84 according to ICD-10)	3	10.34				
Mild (50–69)	11	37.93				
Moderate (35–49)	7	24.14				
Severe (20–34)	8	27.59				
Communication domain (based on Vineland-II communication)	29		31.45	14.01	21	69
Borderline (standard score 70–84 according to ICD-10)	0	0.00				
Mild (50–69)	5	17.24				
Moderate (35–49)	5	17.24				
Severe (20–34)	19	65.52				
**Self-reported QOL (EUROHIS)**	27		4.384	0.59	3	5
**Maladaptive behavior (Vineland-II)**						
Internalizing maladaptive behavior	29		17.138	2.42	13	21
Externalizing maladaptive behavior	29		18.276	2.3	13	24

**Table 3:** Zero-order correlation matrix among intellectual, social and practical functioning variables; quality of life; and maladaptive behavior (N = 29).

	Practical Functioning	Social Functioning	Externalizing Maladaptive Behavior	Internalizing Maladaptive Behavior	Quality of Life
Intellectual Functioning	0.453 *	0.151	0.078	−0.075	0.128
Practical Functioning		0.783 **	−0.019	−0.226	0.292
Social Functioning			−0.218	−0.351 ^†^	0.354 ^†^

Spearman’s correlation coefficient: ^†^ *p* < 0.1; * *p* < 0.05, ** *p* < 0.001.

**Table 4:** Mean differences in self-reported QOL and internal and external maladaptive behavior with and without intellectual disability domain discrepancies (independent samples *t*-test).

	Practical and Social Domains			Intellectual Functioning and Social Domain			Intellectual Functioning and Practical Domain		
	QOL, M (SD)	Internal, M, (SD)	External, M(SD)	QOL, M (SD)	Internal, M(SD)	External, M(SD)	QOL, M(SD)	Internal, M(SD)	External, M(SD)
No Discrepancy	4.381 (0.627)	17.182 (2.462)	18.091 (2.505)	4.597 (0.437)	15.900 (3.071)	17.300 (2.584)	4.506 (0.517)	16.583 (2.999)	18.667 (1.723)
N	21	22	22	9	10	10	11	12	12
Present Discrepancy	4.396 (0.501)	17.000 (2.449)	18.857 (1.464)	4.278 (0.640)	17.789 (1.751)	18.789 (2.016)	4.297 (0.640)	17.529 (1.908)	18.000 (2.646)
N	6	7	7	18	19	19	16	17	17
Mean Difference	−0.015	−0.182	0.766	0.319	1.889 *	1.489	0.209	0.946	−0.667

Self-reported QOL is indicated by a 5-point scale; * = *p* < 0.05; maladaptive behavior is reported as v-scale scores: <18 indicates average level, 18–20 indicates elevated level and 21–24 indicates a clinically significant level.

## Data Availability

Data are not publicly available due to data protection issues.
